# Extensive characterization of NF-κB binding uncovers non-canonical motifs and advances the interpretation of genetic functional traits

**DOI:** 10.1186/gb-2011-12-7-r70

**Published:** 2011-07-29

**Authors:** Daniel Wong, Ana Teixeira, Spyros Oikonomopoulos, Peter Humburg, Imtiaz Nisar Lone, David Saliba, Trevor Siggers, Martha Bulyk, Dimitar Angelov, Stefan Dimitrov, Irina A Udalova, Jiannis Ragoussis

**Affiliations:** 1 Wellcome Trust Centre for Human Genetics, University of Oxford, Roosevelt Drive, Oxford OX3 7BN, UK; 2 Université de Lyon, Laboratoire de Biologie Moléculaire de la Cellule, CNRS-UMR 5239/INRA 1237/IFR128 Biosciences, Ecole Normale Supérieure de Lyon, 46 Allée d'Italie, 69007 Lyon, France; 3 Kennedy Institute of Rheumatology, Imperial College, 65 Aspenlea Road, London W6 8LH, UK; 4 Division of Genetics, Brigham and Women's Hospital and Harvard Medical School, 45 Francis Street, Boston, MA 02115, USA; 5 Harvard-MIT Division of Health Sciences and Technology (HST), Harvard Medical School, Boston, MA 02115, USA; 6 Department of Pathology, Brigham and Women's Hospital and Harvard Medical School, 75 Francis Street, Boston, MA 02115, USA; 7 Université Joseph Fourier - Grenoble 1; INSERM Institut Albert Bonniot, U823, Site Santé-BP 170, 38042 Grenoble Cedex 9, France

## Abstract

**Background:**

Genetic studies have provided ample evidence of the influence of non-coding DNA polymorphisms on trait variance, particularly those occurring within transcription factor binding sites. Protein binding microarrays and other platforms that can map these sites with great precision have enhanced our understanding of how a single nucleotide polymorphism can alter binding potential within an *in vitro *setting, allowing for greater predictive capability of its effect on a transcription factor binding site.

**Results:**

We have used protein binding microarrays and electrophoretic mobility shift assay-sequencing (EMSA-Seq), a deep sequencing based method we developed to analyze nine distinct human NF-κB dimers. This family of transcription factors is one of the most extensively studied, but our understanding of its DNA binding preferences has been limited to the originally described consensus motif, GGRRNNYYCC. We highlight differences between NF-κB family members and also put under the spotlight non-canonical motifs that have so far received little attention. We utilize our data to interpret the binding of transcription factors between individuals across 1,405 genomic regions laden with single nucleotide polymorphisms. We also associated binding correlations made using our data with risk alleles of disease and demonstrate its utility as a tool for functional studies of single nucleotide polymorphisms in regulatory regions.

**Conclusions:**

NF-κB dimers bind specifically to non-canonical motifs and these can be found within genomic regions in which a canonical motif is not evident. Binding affinity data generated with these different motifs can be used in conjunction with data from chromatin immunoprecipitation-sequencing (ChIP-Seq) to enable allele-specific analyses of expression and transcription factor-DNA interactions on a genome-wide scale.

## Background

Single nucleotide polymorphisms (SNPs) that change the pattern of transcription factor (TF) binding to DNA are believed to be a major contributing factor to *cis*-modulation of gene expression; approximately 30% of expressed genes show evidence of *cis*-regulation being influenced by common alleles [1]. In particular, polymorphisms occurring in TF binding sites (TFBSs) that change the pattern of regulatory protein binding to DNA are believed to be a major contributing factor to *cis*-modulation of gene expression. Recent advances in genomic technologies [2-4] are now making allele-specific analyses of expression, TF-DNA interactions and chromatin states possible across the human genome, aiding in evaluation of how DNA polymorphisms in regulatory elements control gene expression.

Chromatin immunoprecipitation-sequencing (ChIP-Seq) and related approaches are now extensively applied to study genome-wide binding of TFs. ChIP-Seq allows the detection of total binding at specific sequences and of their allele-specific activity in cases in which heterozygous sites overlap ChIP-Seq peaks. For example, recent reports extended global allele-specific analysis across individuals to DNA-protein binding [5,6]. Of particular relevance to our study is the work of Kasowski and co-workers [6], in which the authors analyzed binding of the NF-κB protein RELA in stimulated lymphoblastoid cells across eight individuals and documented binding differences between paired individuals at numerous genomic locations.

A major impediment to the ChIP-based evaluation of *cis*-regulatory SNPs is that, by its nature, ChIP can identify genomic regions that interact with TFs but not individual binding sites [7,8]. Other limiting factors in ChIP that can confound measured TF-DNA binding include the state of chromatin at binding regions [9], differing extents of nucleosome occupancy [10], the quality of the antibodies that are so vital to its success and also the near impossibility of isolating a specific dimer instead of all dimers having a subunit in common. Thus, a ChIP-based method is typically used in conjunction with other techniques that can map the site of TF-DNA interactions more precisely. In particular, protein binding microarrays have significantly enhanced our understanding of what individual sequence variants do to alter binding potential within an *in vitro *setting, allowing for greater predictive capability of the effect of a SNP on a TFBS [11-13]. While microarrays were established using a stable attachment of DNA to a solid surface that is in contact with a TF through a liquid medium, other alternative high-throughput platforms, such as Bind-n-Seq [14] or multiplexed massively parallel SELEX (systematic evolution of ligands by exponential enrichment) [8]), are based on both the TF and DNA being in a purely liquid environment. SELEX is a process through which consecutive rounds of selective purification are employed to progressively enrich for a population of DNA ligands that are 'preferentially' bound by the TF in question.

This study focuses on NF-κB, but there is, in general, a great interest within the scientific community to qualitatively and quantitatively define at high resolution all the different DNA sequences bound by TFs [15]. The NF-κB family of TFs has been extensively studied due to its roles in different biological processes like inflammation, apoptosis, development and oncogenesis [16-20]. NF-κB proteins function as homo- or heterodimers, which are made up of Rel homology domain-containing monomers from two subfamilies: the p50 and p52 subfamily (type I subunits); and the RELA, RELB and C-Rel subfamily (type II subunits). Type I subunits lack a transactivation domain and can only activate transcription as a heterodimer with a type II subunit or as a homodimer in complex with co-factors, such as BCL3, IKBZ, and so on [18]. In a given heterodimer, the type II subunit confers transcription-activating capability. Members of the NF-κB TF family bind to a 'core motif' that is between 10 to 11 bases in length [21-23].

**Figure 1:**
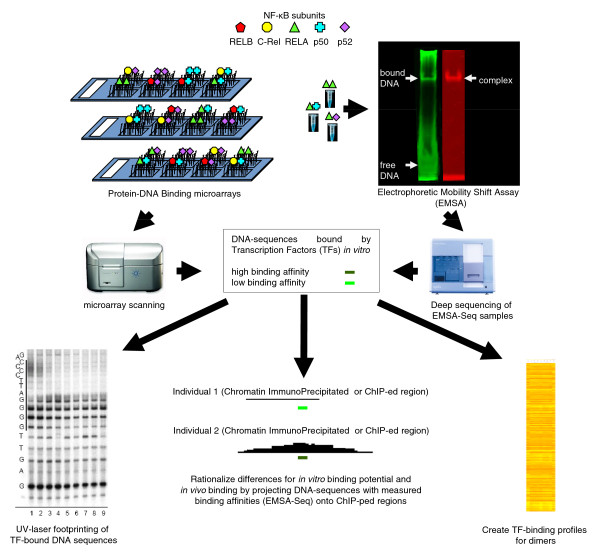
**Outline of the dual platform approach used to profile NF-κB family dimers**. Double-purified, His-tagged NF-κB dimers interact with DNA-probes (microarray) or DNA-ligands (electrophoretic mobility shift assay-sequencing (EMSA-Seq)). Two separate stains are available for the visualization of DNA and protein on EMSA-gels. SYBR Green highlights both DNA bound by the dimer ('bound DNA') and also unbound DNA ('free DNA'). The SYPRO Ruby stain identifies proteins such as those within a dimer-DNA complex ('complex'). Both microarray and EMSA-Seq platforms generate data that provide binding affinities for individual sequences that interact with a dimer. Profiles of nine different dimers illustrating their binding affinities for 803 sequences were constructed using microarrays. In addition, RELARELA, RELAp50 and RELAp52 were also profiled using EMSA-Seq. Deep sequencing revealed dimer-specific binding affinities for distinctive groups of 11-mer sequences. Two classes of these sequences, formed on the basis of similarity to a reference NF-κB binding-model, were used as targets for a UV footprinting experiment. Finally, differences for *in vitro *binding potential as determined using binding affinities from EMSA-Seq and differences for *in vivo *binding as established by a ChIP-Seq study were then co-examined across 7,762 comparisons of paired individuals.

Our overall approach is outlined in Figure 1. We first characterized the binding of nine NF-κB dimers (homodimers of RELA, p50 and p52 and the heterodimers RELAp50, RELAp52, RELBp50, RELBp52, C-Relp50 and C-Relp52) to a limited, 11-mer NF-κB consensus binding space using our microarray platform. This produced data that did not require extensive post-processing and allowed for rapid visualization of the different binding profiles for the dimers. Previously, Badis and co-workers [24] highlighted binding models with coverage of sequence space beyond what has been defined by more canonical models. Included in their study were models with sequence compositions that were again substantially different from those in the canonical models. This suggested that there may be an entire area of 'less canonical' k-mer space that is, as yet, not well defined. We therefore extended our observations to cover this space by further profiling the three RELA dimers using a method we have developed, electrophoretic mobility shirt assay-sequencing (EMSA-Seq) combining EMSA assays done with purified proteins and degenerate oligonucleotide libraries with complete coverage of 11-mer space followed by next generation sequencing of bound DNA molecules. Our results show that a high number of sequences are binders that fall outside of the canonical NF-κB consensus and specificity of binding for typical examples of these novel sequences was validated by UV-laser footprinting.

Finally, we examine the relationships between NF-κB *in vitro *binding affinities (defined as binding potential) and their significance *in vivo *by overlaying sequences and measured binding affinities from our datasets onto genomic locations of RELA ChIP-Seq peaks containing SNPs in stimulated lymphoblastoid cells across eight individuals [6]. Direct positive correlation of NF-κB binding potential with *in vivo *NF-κB binding can be found in 65% of relevant cases examined and these span 1,405 genomic locations that show differences in ChIP-Seq peak heights between individuals. These include regions that may also have potential implications for disease association studies and we show examples in which the risk allele for disease is present in the haplotype associated with higher binding properties *in vitro *and *in vivo*, whereas the normal allele haplotype contains motifs with lower binding properties. This illustrates the utility of studies utilizing TF binding potential for the interpretation of regulatory functional traits.

## Results

### Microarrays show that members of the NF-κB TF family have different binding profiles

To profile DNA binding preferences of multiple NF-κB dimers, double-stranded DNA microarrays containing 803 11-mer sequences within the generalized NF-κB consensus RGGRNNHHYYB flanked by four distinct flanking sequences were hybridized in triplicate with each of the nine recombinant NF-κB dimers (homodimers of RELA, p50 and p52 and the heterodimers RELAp50, RELAp52, RELBp50, RELBp52, C-Relp50 and C-Relp52). A high degree of consistency across experiments was evident given similarity coefficients of at least 0.95 between replicates (Pearson-correlation test).

**Figure 2:**
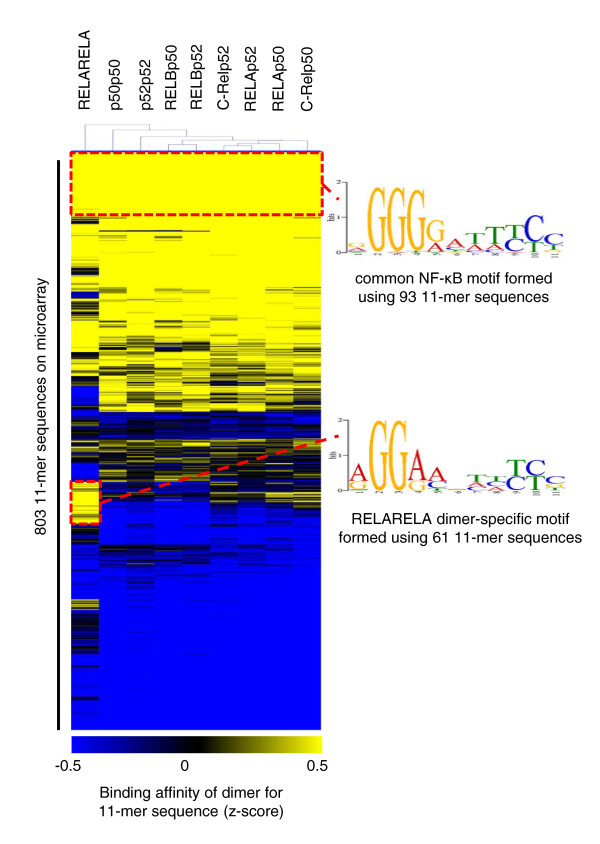
**Binding profiles of the different NF-κB dimers**. Heat map illustration of binding profiles obtained from microarray analysis of dimers. Within the heat map, probes that contain the 803 11-mer sequences and represent 'k-mer' space given by the consensus RGGRNNHHYYB can be found as rows whilst the nine NF-κB dimers have been organized into columns. A graded color scheme has been used to represent the ranked affinities of a dimer for a probe. From lightest to darkest this corresponds to decreasing affinity. Hierarchical clustering was used to describe relationships between binding profiles of the different dimers (Euclidean distance correlation; complete linkage analysis). The profile of RELARELA was largely distinct from those of the other eight dimers. On the whole, homodimers also have binding profiles that render these TFs to be less alike as a class. This is in contrast to the higher degree of similarity found between profiles within the heterodimer class. Two groups of sequences that contribute to similarities and differences between RELARELA and the other dimers have been used to construct representative binding models.

Pair-wise analysis of flank-specific datasets revealed that the binding affinities (*z*-score) of dimers for the 11-mer sequences were largely unaffected by the presence of flanks (Table S1 in Additional file 1). For each probe the median of binding affinities across the four flank-specific datasets of individual dimers was thus used to build representative binding profiles for each dimer (Additional file 2). Pair-wise comparisons of these profiles revealed that the RELA homodimer was most distinct within the entire grouping, with as little as 57% similarity (Pearson-correlation test) to that of the p50 homodimer (Table S2 in Additional file 1). Binding models representing the 50 highest affinity binders were also created for each dimer (Figure S1 in Additional file 1). The use of quantitative data overcomes a known limitation in the classical method of position weight matrix (PWM) construction where individual nucleotide positions within the matrix are assumed to be independent [15]. When the binding data were organized within a heat map and subjected to hierarchical clustering, the profile of RELARELA was clearly distinct from those of the other eight dimers, which was also reflected by the derived binding model for this homodimer (Figure 2). At the same time, there are also elements within the different profiles that are shared across the NF-κB family (Figure 2). On the whole, homodimers had a lower degree of similarity between each other than did heterodimers, with an average similarity coefficient of 0.71 (Table S2 in Additional file 1). Heterodimers, on the other hand, have similarity coefficients averaging 0.95 and tend to recognize DNA sequences in a manner that is more similar to each other (Table S2 in Additional file 1).

### Binding data generated by the EMSA-Seq platform are in good agreement with microarrays

**Figure 3:**
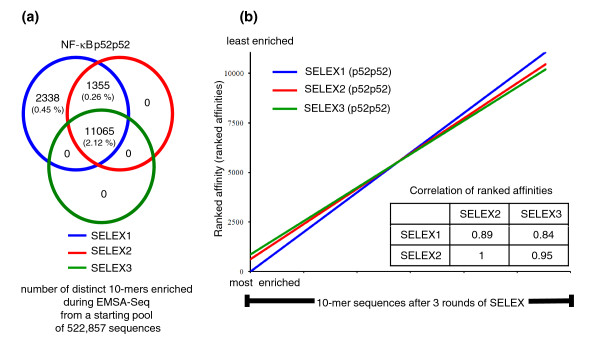
**One round of enrichment was sufficient with NF-kB p52p52**. **(a) **10-mer sequences enriched after one, two and three rounds of selection with NF-kB p52p52 during EMSA-Seq. **(b) **Ranked affinities of 11,065 10-mers that were continually enriched throughout the three rounds of SELEX with p52p52. The correlations of ranked affinities for these sequences throughout the process are shown (Pearson correlation test).

To extend our observations to a substantially larger number of sequences, we then developed a complementary EMSA-seq platform. All sequencing results obtained with this have been deposited into the Gene Expression Omnibus (GEO) database [25] under accession number [GSE:29460]. EMSA-seq employs oligonucleotides containing either 10-mer degenerate regions flanked by a single set of 4-mer sequences (intrinsically comparable to our microarray probes), or a longer 20-mer degenerate region (that is, indirect representation of sequences of different lengths, each one a potential binding site) as DNA ligands in an EMSA assay, followed by DNA extraction, library preparation and deep sequencing of the DNA fraction that has been bound by a transcription factor. To examine the extent of DNA enrichment that is required to generate specific and sensitive binding data, a pool of 10-mer degenerate sequences was subjected to three consecutive rounds of selection by the dimer p52p52. After implementation of quality control measures and a statistical method for determining enrichment, we found that 14,758, 12,420 and 11,065 out of a possible 522,857 10-mer sequences were enriched after one, two and three rounds of SELEX (SELEX1 to SELEX3), respectively (Figure 3a; datasets in GEO under accession number [GSE:29460]). Examination of the non-selected pool revealed that 99.7% of all possible 10-mer combinations were present and this represents a substantial coverage of the entirety of 10-mer space.

In line with reports that an increasingly enriched DNA pool of reduced complexity is typically obtained with more rounds of SELEX [26], we too observed that 25% of sequences identified in the first round were consequently lost after SELEX3 (Figure 3a). The remaining 11,065 sequences were enriched across all three rounds of SELEX and have similarity coefficients of between 0.84 and 0.89 (Pearson correlation tests; Figure 3b). This indicates that SELEX1 would already have revealed the relative enrichment levels for the majority of sequences from SELEX3 (75%) and provides the basis for a single round of enrichment being implemented in EMSA-Seq. Moreover, ligands bound by p52p52 after SELEX1 (Table 1) are substantially less than the 25% of 8-mer sequences thought to be bound specifically by TFs in the study by Jolma and co-workers [8], likely due to an increased presence of non-specific competitor in our TF-DNA binding experiments (see Materials and methods). For these comparisons, we did not perform more than three rounds of SELEX and it is conceivable that the dynamics of TF-binding beyond the third round may be dramatically different from that in preceding rounds. However, this is unlikely given that Jolma and co-workers obtained comparable datasets using between two and four rounds of SELEX [8].

**Table 1:** Comparison and validation of p52p52

	SELEX1	SELEX3
Number/proportion of 10-mer sequences (*n *= 522,857) that were enriched	14,758 (2.8%)	11,065 (2.1%)
Number of 10-mer sequences shared with microarrays (*n *= 757)	249^a ^(32.9%)	196^b ^(25.9%)
Number of 10-mer sequences shared with Linnell *et al. *[13] (*n *= 63)	21^c ^(33.3%)	18^d ^(28.6%)

Hypergeometric probability test for over-representation: ^a^*P *= 6.9e-187; ^b^*P *= 3.1e-148; ^c^*P *= 2.3e-19; ^d^*P *= 1.5e-17. Number of enriched sequences identified during SELEX and overlaps with two microarray datasets (ours and Linnell *et al. *[13]).

Profiling of NF-κB p52p52 from SELEX1 and SELEX3 revealed there was an over-representation of sequences from our arrays and data from Linnell *et al. *[13] (Table 1). In conclusion, the binding data generated by the EMSA-Seq protocol is in good agreement with results obtained using microarrays.

### In-depth profiling of binding specificities of RELA-containing dimers by EMSA-Seq uncovers a binding landscape that extends beyond the known consensus

**Figure 4:**
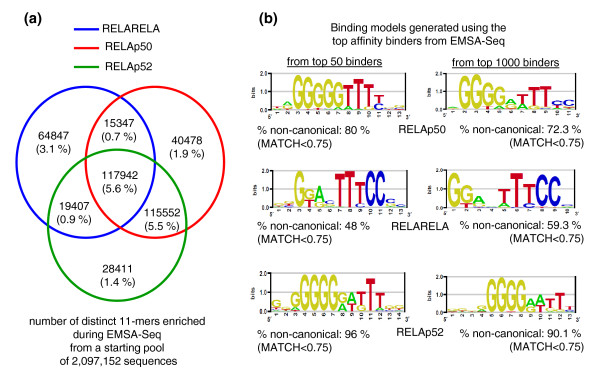
**EMSA-Seq profiling of the NF-κB RELA-containing dimers**. **(a) **Grouping of 11-mer sequences bound by the homodimer RELARELA and the heterodimers RELAp50 and RELAp52 during EMSA-Seq. In parentheses are proportions out of all possible 2,097,152 11-mer sequences. **(b) ***De novo *motif identification was performed on the 50 and 1,000 top-scoring 11-mer sequences from each experiment using the Priority algorithm [51]. No priors were used for motif identification and logos were generated using the enoLOGOS web tool [52]. For every dimer, the percentage proportion of sequences that are non-canonical (MATCH < 0.75) and that have contributed towards construction of the motif has been indicated.

Next, we applied EMSA-Seq to profile binding preferences of three RELA-containing dimers using DNA ligands containing a 20-mer degenerate region and uncovered a rich 'TF-binding landscape' composed of sequences bound with varying affinities. Our deep sequencing approach produced enough data to allow an exhaustive representation of every possible sequence up to a length of 11-mers. Approximately 10 to 13% of all possible 11-mer combinations were bound by each of the three RELA-containing dimers. A breakdown of this is shown in Figure 4a, and datasets have been deposited into the GEO under accession number [GSE:29460]. Binding models representing the 50 and 1,000 highest affinity binders were created for each dimer (Figure 4b). Once again, the profile of RELARELA was distinct from that of the heterodimers RELAp50 and RELAp52 (Table 2). This is consistent with what we observed using microarrays where binding profiles of the two RELA heterodimers are more similar to one another than they are to that of the RELA homodimer (Figure 2).

**Table 2:** Comparison of profiles for RELA-containing dimers

	RELARELA	RELAp50	RELAp52
Proportion of 11-mer sequences shared with RELARELA		61%	63%
Proportion of 11-mer sequences shared with RELAp50			81%
Proportion of 11-mer 'canonical NF-κB binders' (*n *= 4,399) that are enriched	72% (3,167)^a^	84% (3,683)^a^	82% (3,599)^a^
Proportion of enriched 11-mer sequences that have a MATCH score < 0.5	43% (*n *= 217,543)	47% (*n *= 289,319)	61% (*n *= 281,312)

Similarities between the binding profiles of the three dimers with proportions of 'canonical NF-κB binders' and sequences with MATCH scores < 0.5 present in each. ^a^Hypergeometric probability test for over-representation: *P *= 1e-99.

Binding sequences can be categorized on the basis of similarity (MATCH score) to a reference binding model, either an established PWM or an alternative constructed from quantitative data (Table S3 in Additional file 1). We created two sets of MATCH scores for 11-mer sequences in our microarray and EMSA-Seq datasets, one based on the reference binding model and another on the alternative formed using the 300 highest affinity binders from our EMSA-Seq data (see Materials and methods and Supplementary Material in Additional file 1). Both are highly comparable, with 95% similarity between the two sets (Pearson correlation test).

For subsequent analysis, we also defined a group of 4,399 11-mer sequences termed 'canonical NF-κB binders', computationally derived on the basis of a greater than 0.75 MATCH score similarity to the canonical NF-κB PWM (Additional file 3). These were over-represented in our EMSA-Seq datasets and many would be recognized as being familiar targets of NF-κB (Table 2). One of the most intriguing observations from this study is that some of the most enriched sequences do fall outside of the known NF-κB consensus space (Table 2). Examples of such non-canonical sequences include AGGGGGATCTG, AGGGAAGTTA and CTGGGGATTTA. MATCH scores of 0.49, 0.43 and 0.29, respectively, render these three sequences quite different from the generalized 11-mer consensus RGGRNNHHYYB.

### Non-canonical sequences identified in EMSA-Seq exhibit specific binding by UV laser and DNaseI footprinting

To further examine the interactions of NF-κB dimers with these non-canonical sequences that are different to the reference, we used DNase I and UV laser footprinting combined with EMSA techniques. As a positive control, we studied the binding of NF-κB dimers to two known NF-κB binding sequences, H-2 (GGGGAATCCCC) and HIV (GGGGACTTTCC).

**Figure 5:**
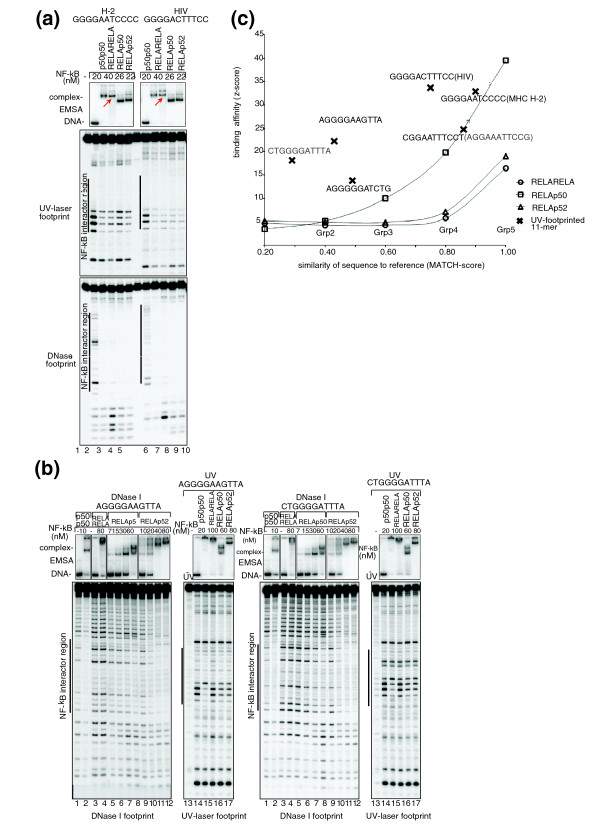
**Specific interaction of NF-κB dimers with canonical and non-canonical sequences**. **(a) **Interaction of four NF-κB dimers, p50p50, RELARELA, RELAp50 and RELAp52, with canonical sequences containing either a H-2 binding site (lanes 1 to 5), or a HIV recognition site (lanes 6 to 10). These were profiled using EMSA (top panel), UV laser (middle panel) and DNAse I (bottom panel) footprinting techniques (with interactor regions demarcated with vertical black lines). For example, RELA dimer-DNA complexes were detected with EMSA (lanes 3 and 8; red arrows). Furthermore, a 'UV footprint' in the form of lower intensity banding observed within the interactor region (relative to controls in lanes 1 and 6) indicates specific interactions of varying affinities between the dimer and DNA. **(b) **Interaction of RELARELA with the non-canonical sequences was non-specific. With both sequences, distinct dimer-DNA complexes were observed by EMSA with all dimers except RELARELA, for which a smear was obtained (lane 4: RELARELA). No footprint was observed with RELARELA, whilst for the other dimers a stronger footprint was obtained with AGGGGAAGTTA compared to CTGGGGATTTA. **(c) **Median enrichment of 11-mers bound by the three RELA-containing dimers in EMSA-Seq. Five groupings of sequences were formed on the basis of MATCH similarity (Grp1 ≤ 0.20, 0.201 ≥ Grp2 ≤ 0.40, 0.401 ≥ Grp3 ≤ 0.60, 0.601 ≥ Grp4 ≤ 0.80 and Grp5 ≥ 0.801). There is a trend of enrichment increasing alongside MATCH similarity. Also shown are the average enrichment values and corresponding similarities to the reference for the six 11-mer sequences that were footprinted (crosses with sequence indicated).

EMSA with the p50p50 and RELA homodimers, RELAp50 and RELAp52, was first used to establish that a dimer-DNA complex was formed, which was subsequently studied using DNase I and UV laser footprinting. These two techniques identify the specific binding of a dimer to a DNA sequence in the form of a signature or 'footprint' of reduced intensity at binding regions. DNase I footprinting allows one to qualitatively distinguish between specific and non-specific binding, while UV laser footprinting works on the principle of dimer-DNA complexes being irradiated by a single UV laser pulse followed by mapping of the induced photo lesions at 1-bp resolution. It has the added capability of quantifying the strength of a dimer-DNA interaction (binding constant K_d_). Both H-2 and HIV sequences produced strong and specific binding patterns with the different dimers tested (Figure 5a).

Next, we determined by UV laser footprinting the binding affinities of the three RELA-containing dimers for one canonical, AGGAAATTCCG, and three randomly selected non-canonical sequences (the three examples described in the previous section). We cross-compared these results with those from the microarrays and EMSA-Seq (Table 3). The canonical AGGAAATTCCG sequence was bound by the RELA homodimer in all assays. Interestingly, all three non-canonical sequences, AGGGGGATCTG, AGGGAAGTTA and CTGGGGATTTA, were not specifically bound by this same homodimer. Correspondingly, RELARELA also either did not bind these sequences in EMSA-Seq or bound them with only low affinity. In contrast, specific dimer-DNA interactions occurred between the RELA heterodimers and non-canonical sequences (Figure 5b), in agreement with EMSA-Seq data (Table 3). Thus, we concluded that the binding of selected NF-κB dimers to non-canonical sequences was indeed specific. Importantly, whilst our data show that there is the overall tendency for sequences with higher MATCH scores to be bound by a TF with higher affinities (Figure 5c), there is also variation in affinities amongst sequences with comparable MATCH scores (Figure S2 in Additional file 1).

**Table 3:** Binding affinities of RELA-containing dimers for canonical and non-canonical sequences

		RELARELA			RELAp50			RELAp52		
		Binding affinity (*z*-score)		Binding affinity (K_d_)	Binding affinity (z-score)		Binding affinity (K_d_)	Binding affinity (z-score)		Binding affinity (K_d_)
11-mer sequence	MATCH_score	Microarray	EMSA-Seq	UV-laser footprint	Microarray	EMSA-Seq	UV-laser footprint	Microarray	EMSA-Seq	UV-laser footprint
AGGAAATTCCG	0.86	3.70	40.90	3.25	1.20	20.42	4.60	0.55	13.00	1.70
AGGGGGATCTG	0.49	Non-binding	Non-binding	Non-binding	2.39	23.10	10.50	1.76	18.35	2.00
AGGGGAAGTTA	0.43	NA	3.78	Non-binding	NA	35.41	26.00	NA	27.50	20.00
CTGGGGATTTA	0.29	NA	10.84	Non-binding	NA	24.17	16.00	NA	19.54	13.80

Binding affinities were measured using microarrays, EMSA-Seq and UV laser footprinting. Canonical sequences have MATCH scores ≥ 0.75 whilst non-canonical sequences have MATCH scores < 0.75. Where a sequence was not present on the microarrays this has been indicated with 'NA'. Decreasing binding affinities correspond to decreasing *z*-scores for both microarrays and EMSA-Seq, but increasing K_d _values in the case of measurements done with UV laser footprinting. All values were derived from three and two independent experiments for microarrays and UV laser footprinting, respectively. Values for EMSA-Seq were derived from datasets obtained from the pooling of three independent experiments per dimer.

### Examining NF-κB activity *in vivo *using data from DNA-binding platforms

**Figure 6:**
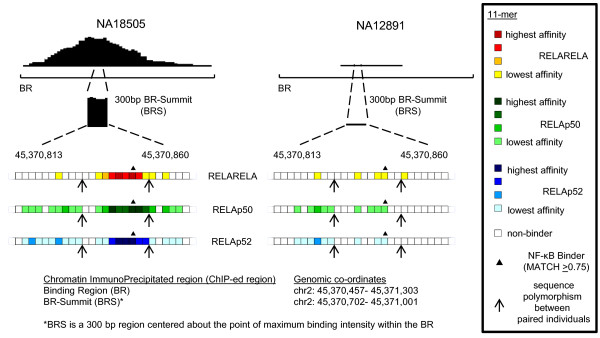
**Direct positive correlation of binding potential and *in vivo *binding**. Presence of dimer-specific 11-mer sequences (colored boxes) enriched during EMSA-Seq within a 300-bp region (BRS) inside a binding region (BR) that was isolated during immunoprecipitation of RELA. Boxes are 11-mer sequences shown with an overlap of 10 bp for adjacent boxes. The gradient of coloration within boxes corresponds to relative binding affinities as determined by EMSA-Seq. A non-colored box represents an 11-mer sequence that was not bound by a RELA-containing dimer. Sequences that are also known NF-κB binders are indicated (filled triangles). Arrows indicate the positions of sequence polymorphisms between the two individuals.

To estimate the NF-κB binding potential as measured by EMSA-Seq for the interpretation of *in vivo *NF-κB binding, we overlaid dimer-specific 11-mers from our datasets onto all binding region summits (BRSs; see Materials and methods) from a study by Kasowski and co-workers [6]. In effect, 11-mer binders identified by EMSA-Seq were mapped onto a 300-bp region, the BRS, which is centered on the summit point within a binding region (BR) (Figure 6). For visualization purposes, the intensity of the coloration used during mapping is reflective of the binding affinity of a NF-κB dimer for 11-mer sequences identified by EMSA-Seq. The NF-κB binding potential of a BRS was then calculated by adding up the *in vitro *binding affinities of a set of dimer-specific 11-mers, either the homodimer or a heterodimer of RELA. Using data from the 1000 Genomes Project, we identified polymorphisms, if any, within the BRSs of paired individuals. Polymorphisms may or may not alter the composition of 11-mer sequences within the BRS of an individual. For example, as a direct consequence of two polymorphisms, individual NA18505 has higher NF-κB binding potential compared to individual NA12891 and this corresponds to a greater extent of *in vivo *NF-κB binding observed (Figure 6).

Kasowski and co-workers [6] determined that a total of 25,764 comparisons had differences in NF-κB binding between paired individuals. Our analysis revealed that of these, only 7,762, covering 2,710 BRSs, are associated with paired individuals having sequence polymorphisms within the BRS. This is an important point as only in this subset of comparisons can differences in NF-κB binding between paired individuals be directly attributed to differences in DNA sequence. Using our data in conjunction with these comparisons, we sought to generate an 'extended NF-κB binder' set of 11-mers defined on the basis of enrichment during EMSA-Seq, but also taking into account similarity to the reference binding model. Estimations of *in vitro*-*in vivo *correlation made using the 5,000 most enriched sequences were considerably more successful (71% direct positive correlation; Figure S3a in Additional file 1) than those with the 5,000 least enriched sequences (51% direct positive correlation; Figure S3a in Additional file 1). A direct positive correlation is when the trend of binding differences for *in vivo *binding and *in vitro *binding potential (EMSA-seq) is in the same direction across paired individuals. It is also striking that with the exclusive use of binding potentials derived from a subgroup of highly enriched sequences that are not within the defined 'canonical NF-κB binders' subset, we were still able to achieve 71% *in vitro*-*in vivo *correlation (Figure S3b in Additional file 1). Our optimal result was achieved using only 11-mers enriched at levels greater than the median *z*-scores for specific sets or 'bins' of sequences formed on the basis of MATCH scores (minimum of no less than 10% below median value for each MATCH score 'bin'; Figure S3c in Additional file 1). This included all the enriched sequences that also interacted specifically with the RELA-containing dimers as judged by footprinting (Figure 5c) and allowed for the investigation of 5,452 comparisons covering 1,959 BRSs, in essence representing the best compromise between sensitivity and accuracy for *in vivo*-*in vitro *comparisons. Direct positive correlation of *in vitro *NF-κB binding potential with *in vivo *NF-κB binding was observed in 3,559 comparisons covering 1,405 BRSs (or 65% of 5,452 comparisons). There are 1,893 comparisons covering 883 BRSs (or 35%) that displayed no direct correlation between *in vitro *and *in vivo *data, and there are 2,310 (958 BRSs) comparisons in which genomic variation between individuals has not resulted in any detectable difference in binding potential, due to SNPs either not affecting 11-mers within our datasets or affecting only very low affinity binders (Figure S4 in Additional file 1).

**Figure 7:**
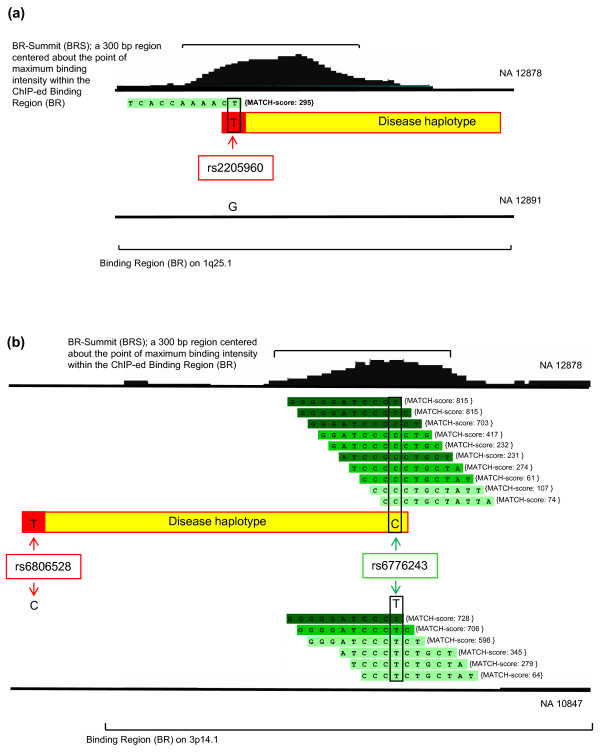
**Binding potential and risk alleles of disease**. **(a) **The trait/disease-associated (TAS) SNP rs2205960 (box, red outline) is associated with systemic lupus erythematosus. The 300-bp region (BRS) of individual NA12878, who is a carrier of the risk allele (T), contains a single 11-mer that was enriched during EMSA-Seq with RELAp50 whereas NA12891, who carries the normal allele, does not. This has resulted in NA12878 having a higher NF-κB binding potential that is directly correlated to higher *in vivo *binding. **(b) **The TAS rs68065278 (box, red outline) is associated with celiac disease. It is in linkage disequilibrium with another polymorphism, rs6776243, present within the BRS (box, green outline). The BRS of individual NA12878, who is a carrier of the C allele for rs6776243, contains more 11-mers that were enriched during EMSA-Seq with RELAp50 than that of the other individual. This has resulted in NA12878 having a higher NF-κB binding potential that is likewise directly correlated to higher *in vivo *binding.

Using the 3,559 comparisons covering 1,405 BRSs for which there is direct positive correlation, we examined potential implications for disease association studies. From a database listing all genome-wide association studies (GWASs) [27], we created a comprehensive list identifying the trait/disease-associated SNPs (TASs) having the highest risk association within each study (Additional file 4). All 3,407 TASs analyzed were mapped to the nearest BRS. We focused on TASs within 1 kb from the center of the nearest BR (this region is also referred to as a BRS) to ensure good linkage disequilibrium between the TAS and a SNP under the peak. From all TASs identified in the database, 13 were within this limit and from these we observed a prevalence of inflammatory disease-associated polymorphisms, in particular those linked to autoimmune diseases (8 of 13 TASs with *P *= 2.8e-05; hypergeometric probability test for over-representation). We present two examples of this. In Figure 7a, TAS rs2205960 is a SNP that is within the BRS, and not only is the disease allele (T) associated with systemic lupus erythematosus, but according to our data it creates a potential binding site which in turn is associated with increased *in vivo *RELA binding. In another case (Figure 7b), TAS rs6806528 has been described as being associated with celiac disease; this TAS is found within the BR, not the BRS, but more importantly it is in perfect linkage disequilibrium with another SNP that is under the BRS, rs6776243. The disease allele (rs6806528, allele T) thus segregates perfectly with the allele associated with both high *in vivo *binding and *in vitro *binding potential (rs6776243, allele C). In both cases, the risk allele for disease is present in the haplotype associated with higher *in vivo *binding and also higher *in vitro *binding potential, whereas the other haplotype containing the normal allele is associated with lower *in vivo *binding and lower *in vitro *binding potential.

## Discussion

Eukaryotic genes are regulated largely through the interactions of TFs and their assembly into enhancer complexes. There are many examples of DNA variation in enhancers that affects transcription factor binding and has functional consequences for gene expression, for example in the NF-κB and OCT1 sites in the tumor necrosis factor promoter [28,29]. Our earlier studies aimed to predict *in silico *the effects of SNPs within regulatory sequences using a statistical model to describe NF-κB-DNA interactions [21]. Here we applied a novel experimental approach to profile NF-κB DNA binding properties *in vitro *(but which is applicable to any other TF) and documented differences in binding preferences between the various protein dimers. We then used our data to explain differences in *in vivo *NF-κB recruitment between eight individuals [6]. We achieved a significant increase in the number of interpretable effects compared to when only canonical motifs were considered and also observed an association between TF binding and the allelic signature for disease.

### Profiles of binding affinities built using this dual-platform approach (microarrays and EMSA-Seq)

The correlation of binding data generated by both EMSA-Seq and microarray platforms is in the order of 77 to 84% (Figure S5 in Additional file 1), indicating that they cross-validate each other well. Using both platforms we observed that the RELARELA homodimer was most distinct from the other dimers (Figures 2 and 4a), a finding confirmed by DNaseI and UV laser footprinting. On the other hand, the binding profiles of heterodimers containing p50 or p52 subunits were more similar (Table 3). These results agree with the findings of Chen and co-workers [30,31], who showed that DNA sequences bound by RELARELA were distinct from those bound by homodimers of p50 and p52. The GGAA motif was strongly associated with RELA-bound sequences whilst GGGRY was more prevalent in sequences bound by p50 and p52. Indeed, we found that within the 100 11-mer sequences for which RELARELA had the highest affinity, 76% of these contained a GGAA motif whilst only 42% contained a GGGRY motif. This is manifested in a representative binding model for RELARELA built using 61 sequences that were preferentially bound by this dimer only (Figure 2). Conversely, with RELAp50 and p50p50, only 37 to 47% of the 100 sequences for which they had the highest affinity contained a GGAA motif, whilst 64 to 67% of these sequences contained a GGGRY motif. Our results support the hypothesis that p50 and p52 subunits have a major influence on the binding characteristics of NF-κB dimers (Figures 2 and 4a; Table 3). Of interest, in agreement with Badis and co-workers [24], we observed that lower affinity sequences contributed most to dimer-specific preferences. Two of the proteins in our study, RELAp50 and RELBp52, are activated by distinct NF-κB pathways within the cell, the canonical and alternative, respectively. Interestingly, two previous studies examining the binding characteristics of these TFs reached different conclusions. In their approach using 200 sequences containing 10-mer motifs derived from random site selection, Britanova *et al. *[32] reported a lack of distinction in the binding of these two heterodimers. This contradicts a previous report by Bonizzi *et al. *[33] in which these two heterodimers could recognize distinct motifs. In particular, the sequence GGGAGATTTC present at the B-Lymphocyte Chemoattractant (BLC)-κB binding site, for which Britanova *et al. *could not detect any binding. Overall, our data agree with Britanova *et al. *in that we did observe a 95% correlation in the binding properties of these two dimers (Table S2 Additional file 1). On the other hand, we also identified RELBp52 as being the stronger binder of the BLC-κB sequence and this is in agreement with Bonizzi *et al *(microarray data provided as Additional file 2). The discrepancy concerning specifically this sequence may have arisen from differences between protein preparations (mammalian- versus bacteria-based systems) or experimental conditions.

We feel that whilst our profiling, done using microarrays that encompass a comparatively larger number of sequences, has shown that RELBp52 has an overall similar binding profile to RELAp50, individual sequences did show distinct binding properties between the two proteins. Thus, an exhaustive profiling of these dimers using EMSA-Seq would be a logical follow-on to this study, which should then give us more insight into their binding preferences.

### Optimal interpretation of NF-κB DNA binding requires both canonical and non-canonical sequences

A recent study examining the relationship between protein binding microarray-derived binding-models and *in vivo *binding had to make an assumption that the experimentally derived affinities of DNA sequences were equally applicable to binding *in vivo *[7]. Analyzing the relationship between DNA sequence and binding using a dataset derived from several individuals offers the advantage of examining binding between individuals at the same genomic location rather than across different ones. Data from the 1000 Genomes Project coupled with those from Kasowski and co-workers have enabled us to compare differences in binding between individuals across the same genomic locations but that had polymorphisms in DNA sequence. Our set of 'extended NF-κB binders' provided the optimal compromise between sensitivity and accuracy for estimation (Figure S3c in Additional file 1). When we interrogated the same data using only our measured binding affinities obtained for 3,109 of the 4,399 'canonical NF-κB binders', we could visualize differences across only 892 comparisons covering 276 BRSs with direct positive correlation in 82% of the data (Figure S6 in Additional file 1). Whilst this is comparable to a computationally derived result of 79% in yeast [4], it also clearly demonstrates the limitation of canonical NF-κB binding motifs in interpreting more than a small fraction of binding events overall. For example, 1,273 BRSs do not involve 'canonical NF-κB binders' in any pair-wise comparison but do include 'extended NF-κB binders'. Our data thus provide strong evidence for the value of *in vitro*-enriched k-mers in estimating NF-κB binding potential and emphasize the positive contribution of non-canonical binders outside of the classical NF-κB consensus (Figure 6; Figure S3b in Additional file 1).

Of interest, despite us having profiled three RELA-containing dimers, there are still 1,893 comparisons covering 883 BRSs for which there is no direct correlation between binding potential and *in vivo *binding (Figure S3c in Additional file 1). Immunoprecipitated TFs are often part of larger protein complexes and identifying direct binding to DNA is therefore no trivial task in these cases [34,35]. It is possible, therefore, that for the comparisons where we were unable to correlate NF-κB binding potential with *in vivo *NF-κB binding, RELA may not have bound directly to DNA. By mapping TASs within the BRS, we observed that there was a high prevalence of inflammatory disease-associated polymorphisms. This includes auto-immune conditions, such as celiac disease [36], systemic lupus erythematosus [37], primary biliary cirrhosis [38], rheumatoid arthritis [39], Crohn's disease [36], multiple sclerosis [40] and also a trait associated with immunoglobulin A deficiency [41]. The inflammatory response, of which NF-κB is a key modulator, features prominently in all of the above mentioned conditions. As a ubiquitously expressed TF, NF-κB plays a major role in many biological processes, namely inflammation and immunity. Upon activation, NF-κB translocates to the nucleus and binds specific motifs within the genome in order to activate transcription of genes associated with these and other processes. We used a database of disease-associated polymorphisms and identified 13 SNPs, all present within 1 kb of the BRS, of which 8 were linked to inflammatory diseases. It is reasonable to hypothesize then that increased NF-κB binding observed at BRs associated with disease traits may contribute to increased levels of inflammation and immune activity.

Our design for EMSA-Seq included a deep sequencing approach, which allowed for an extensive survey of sequences bound by TFs far beyond the numbers achieved by methods such as standard SELEX, and provided the statistical power to discriminate enriched sequences from background. Our deep sequencing approach yielded an average of 6 million reads per experiment, with the highest being 15 million, in contrast to 30,000 to 300,000 reads obtained in experiments with a single TF in two other comparable methodologies [8,14]. Despite this, there remained 2,310 comparisons in which genomic variation has apparently not resulted in any difference in binding potential between individuals (see Results). To address this, deeper sequencing may be required in order to both identify other binders and enhance perception of differences in binding affinities between binders, thus offering invaluable insights into the strengths and limitations of different implementations of EMSA-based technologies.

## Conclusions

With our data we were able to describe differences in binding preferences between NF-κB dimers. We showed that NF-κB binds not only canonical but also non-canonical motifs and generated data that greatly enhances our ability to describe NF-κB binding sites. This facilitated the analysis of NF-κB binding sites throughout the genome, revealing SNP variation between individuals. Through this we were able to determine the effect of SNPs on NF-κB binding. This study represents a major development in interpreting data generated by techniques like ChIP-Seq, as well as expression quantitative trait loci (eQTL) data and variations reported in GWASs of functional traits. NF-κB is only one among scores of common TFs that regulate a majority of genes. It should be feasible, therefore, to generate similar data for other TFs to interpret and predict the effects of variations on TF binding genome-wide and to begin to model how gene expression varies as a function of polymorphisms within binding sites.

## Materials and methods

Sequences of the different primers and DNA ligands can be found in Additional file 5. All quantification of nucleic acid samples was performed according to manufacturer instructions on a Qubit Fluorometer (Invitrogen #Q32857, Paisley, United Kingdom) and with either the Quant-iT dsDNA High Sensitivity Assay Kit (Invitrogen #Q33120) or the Quant-iT dsDNA Broad Range Assay Kit (Invitrogen #Q33130). Protein assays were performed using the Quant-iT™ Protein Assay Kit (Invitrogen #Q33210).

### Protein expression and purification

Expression constructs for the nine NF-κB dimers (*Homo sapiens*) used in this study were created following a set of procedures previously established by Udalova and co-workers [42]. The dimers are formed from these subunits: RELA (p65), RELB, C-Rel (REL), p50 (NFKB1) and p52 (NFKB2). Briefly, pET vectors for expression in BL21 (DE3) *Escherichia coli *(Merck, Nottingham, United Kingdom) were used to produce histidine-tagged (His-tagged) recombinant proteins. Proteins were over-expressed through induction with 0.2 mM isopropyl β-D-1-thiogalactopyranoside (IPTG) at 30°C for 5 hours. Pellets of cells were harvested in 'Ni-NTA binding' buffer with added EDTA-free protease inhibitor (Roche, West Sussex, United Kingdompulse-sonicated for 2 minutes and debris removed via centrifugation at 16,000 *g*. A two-step purification procedure was then employed, first with the 'Ni-NTA His-Bind Resin' system (Merck #70666) and then a subsequent purification based on DNA-affinity isolation of functional, DNA-binding protein. Ni-NTA purification was carried out according to the manufacturer's guidelines. For DNA-affinity isolation, the processing of a sample derived from 250 ml of bacteria culture required 0.128 μM of oligonucleotides comprising the TNF promoter (biotinylated) and complementary sequence of this. Prior to use, the oligonucleotides were annealed via incubation in NEB Buffer 3 at 94°C for 1 minute then subsequently for an additional 69 cycles of 1 minute each coupled to a per-cycle, step-wise decrease of 1°C. A pre-annealed oligo mixture (712.5 μl) was conjugated with streptavidin-agarose (Sigma, Dorset, United Kingdom) before once-purified material from the preceding step was added to it.

### Protein binding microarrays

We designed 8 × 15 K Agilent arrays using eArray [43] (details can be found in Additional file 6). Briefly, using the canonical consensus sequence GGRRNNYYCC [13,23] as a start point, we expanded this in order to represent not only the strict consensus but also a host of other sequences. Our expanded 11-mer motif, RGGRNNHHYYB, was processed using the principal co-ordinates method developed by Udalova and co-workers [21]. The outcome was 803 DNA sequences that are representative of the 'k-mer space' encompassed by the expanded motif. Our microarray covers relatively little of '11-mer space' when compared to another previously described by Berger and co-workers [44] that has exhaustive coverage of '10-mer space'. However, our uncomplicated probe-design with its well-defined regions has the advantage of fewer confounding factors when interpreting TF-DNA binding events. For example, only one variable region 11-mer is present on a probe (Additional file 6). In addition, four different configurations of each 11-mer, as determined by the flanking sequence around it, are represented on four separate sets of probes. Protocols for the preparation and hybridization of microarrays can be found in Additional file 1.

### EMSA-Seq (TF-DNA binding followed by EMSA and deep sequencing)

We chose DNA ligands that were 60-mers in length to facilitate both the formation of double strands and the library preparation procedure that precedes sequencing. Protocols for the creation of double-stranded oligonucleotide pools and preparation of libraries for deep sequencing can be found in Additional file 1.

For TF-DNA binding followed by EMSA, essentially a 20-μl reaction composed of purified protein, double-stranded DNA ligand and 125 μg/μl poly dI-dC buffered in 12 mM HEPES pH7.8, 75 mM KCl, 1 mM EDTA, 4 mM GTP and 12.5% glycerol was incubated at room temperature for 1 hour. A DNA:protein ratio of 8:1 molecules was maintained in our TF binding experiments. TF-DNA mixtures were subsequently loaded onto a 6% DNA retardation gel (Invitrogen #EC6365BOX) alongside 'no protein' and 'no DNA' controls, and migrated in Novex 0.5 × TBE running buffer (Invitrogen #LC6675) for 1.5 hours. Gels were stained using Invitrogen's EMSA-Kit (#E33075) following the manufacturer's procedures. Visualization of gels was performed using either a DR46B Transilluminator (Clare Chemical Research, Dolores, Colorado, USA or LAS-4000 system (Fujifilm, Japan). Bands containing TF-DNA complexes were excised from gels, elution of DNA carried out overnight at room temperature using a diffusion buffer (0.5 M ammonium acetate, 10 mM magnesium acetate, 1 mM EDTA pH 8.0, 0.1% SDS) and the DNA purified using QIAGEN's polyacrylamide gel extraction protocol (#20021) (QIAGEN, West Sussex, United Kingdom). This was then processed for deep sequencing (Supplementary Material in Additional file 1).

For experiments in which several rounds of SELEX were carried out, the following procedure was adopted. DNA obtained after the first round of TF-DNA binding followed by EMSA, elution and purification was amplified using a high-fidelity PCR procedure adapted from Beinoraviciute-Kellner and co-workers [45]. Briefly, multiple 100-μl reactions, each composed of 0.2 mM dNTPs, 1 μM primers 1 and 2, together with 2 units of KOD Hot Start Polymerase (Merck #71086-3) buffered in accompanying 1 × PCR buffer, were incubated for 15 s at 95°C, then subjected to 25 PCR cycles (5 s 95°C, 5 s 60°C, 5 s 70°C). DNA was pooled, purified using phenol-chloroform and concentrated via ethanol precipitation. This was subsequently used as starting material for TF-DNA binding in the second round of SELEX.

### EMSA, DNase I and UV laser footprinting

High performance liquid chromatography-purified oligonucleotides containing NF-κB binding sites were purchased from MWG (Ebersberg, Germany) and further processed into labelled probes as described in the Supplementary Material in Additional file 1.

TF-DNA binding reactions were then prepared in buffered volumes of 25 μl (10 mM Tris, pH 7.4, 75 mM NaCl, 1 mM EDTA, 1 mM dithiothreitol, 200 μg/ml bovine serum albumin, 0.005% NP-40). From this, an aliquot of 5 μl was used for EMSA, 10 μl for DNase I footprinting as previously described [46] and the remainder for UV laser footprinting, which involved exposure to a single, high intensity UV pulse from the fourth harmonic generation of a nanosecond Nd:YAG laser (wavelength, 266 nm; pulse duration, 5 ns; energy, 0.1 J/cm^2^; Surelite 1, Continuum USA, Villebon sur Yvette, France). DNA was then supplemented with 0.1% SDS, purified by phenol-chloroform extraction, ethanol precipitated, dissolved in the binding buffer and thoroughly digested using Fpg protein and T4 endonuclease V (Trevigen, Montucon, France) for 30 minutes at 30°C. This was then resuspended in a formamide loading buffer and migrated on a 13% polyacrylamide sequencing gel. Dried gels were exposed overnight on a phosphorimager screen and the images analyzed using a Fuji 5100 Phosphorimege scanner and Multi Gauge 3.0 software (Fujifilm). Affinities (K_d_) of the different dimers for binding sites represented on each probe were determined using the following procedure. Intensities of guanine (8-oxoG) and pyrymidine (cyclobutane pyrimidine dimers) 'cleavage bands' within the binding site were quantified by integration and normalized to either total radioactivity loaded or a reference guanine 'cleavage band' located outside of the binding site. Curves representing normalized cleavage band intensities versus dimer-concentration were 'least square deviation fitted' by smooth dependencies, and K_d _was determined as dimer concentration corresponding to half of the amplitude change.

### Statistical analyses

#### Data pre-processing

For microarrays, a *z*-score was obtained using log_2_-transformed intensities and the median of replicates calculated for each probe within every array. EMSA-Seq involved establishment of enriched 10- and 11-mer sets corresponding to selection by the dimers p52p52, RELARELA (p65p65), RELAp50 (p65p50) and RELAp52 (p65p52). The processing of reads obtained after deep sequencing is described in the Supplementary Material in Additional file 1. All 'Meryl' k-mer counts for a sequence obtained from these processed reads have been normalized against the dataset with the lowest number of acceptable reads for that sequence.

A binomial distribution model was used to determine enrichment (*z*-score) of 'Meryl' k-mers for all datasets. To determine which 10- or 11-mers were significantly enriched (10-mers used in p52p52 experiments, 11-mers for everything else) as a result of the selection process, the number of Meryl 10- or 11-mers obtained after protein selection was compared against the number of Meryl 10- or 11-mers generated by the sequencing of a control pool. To this end, we modeled the number of times each 10- or 11-mer is observed in a pool as a binomial distribution with parameters 'n' and 'p'; 'n' corresponds to the total number of 10- or 11-mer observations in the pool and 'p' the probability of observing a given 10- or 11-mer. In the absence of selection this probability is assumed to be identical for both the control and protein-selected pools. If, on the other hand, the 10- or 11-mer is preferentially bound by the protein, the probability of observing this 10- or 11-mer is then expected to be increased in the protein-selected pool. These two scenarios are compared through a likelihood ratio test and *P*-values are corrected for multiple testing using a Benjamini-Hochberg procedure to enforce a false discovery rate of 0.01. Fundamentally, a 10- or 11-mer that is highly enriched after selection (high *z*-score) and has a low, corrected *P*-value is one that is bound by a protein at high affinity (Figure S7 in Additional file 1).

#### Over-representation of a category within datasets

Hypergeometric probability tests were used to test the significance of categories within datasets.

### Analyses of enriched 10- or 11-mers

#### Mapping of 11-mers within BRs, derivation of NF-κB binding potential and determination of direct positive correlation between binding potential and *in vivo *TF binding

Scripts were used to map the presence and location of 11-mers within a 300-bp segment centered about the position within the BR that has the maximum number of ChIP-Seq tags (these are BRSs). For our analyses we have established the genomic coordinates of a BRS to be BR-specific and these do not vary between different individuals. Mapping was performed for all BRSs across eight individual genomes.

Two methods to determine overall NF-κB binding potential for a BRS were tested. First, z-scores for all enriched 11-mers from the three RELA-containing dimer datasets that could be mapped within a BRS were added up. A difference, if any, in binding potential between BRSs of paired individuals was then determined. Between paired individuals, a 'successful' rationalization of binding potential and *in vivo *TF binding (direct positive correlation) was when the trends of difference in both were in the same direction. Second, z-scores for all enriched 11-mers from one RELA-containing dimer within a BRS were added up. For pairwise comparisons that were not successfully rationalized, the process was repeated with a different RELA-containing dimer until all datasets had been covered. The optimal result was given by the second method.

#### Use of the tool MATCH as a basis for similarity to a reference binding model

MATCH as implemented by Kel and co-workers [47] was used for the derivation of a similarity score for 11-mer sequences in relation to a reference binding model. Two sets of MATCH scores have been assigned to 11-mer sequences (Additional file 7). One set was derived using a binding model based on V$NFKB_Q6_01, a PWM for NF-κB in the TRANSFAC database (this is also our reference binding model; Table S3 in Additional file 1), and the other on the 300 highest affinity binders from all three RELA-containing dimer EMSA-Seq datasets (this is an alternative binding model created using our EMSA-Seq binding data; Table S3 in Additional file 1). Theoretically, a MATCH score of 1.0 corresponds to the highest degree of similarity possible whilst 0 corresponds to the lowest.

#### 'Canonical NF-κB binders', a group of 11-mer sequences with high similarity to V$NFKB_Q6_01

A grouping of 4,399 sequences termed 'canonical NF-κB binders' was formed by using all sequences with MATCH scores > 0.75 (based on reference binding model). These can be found in Additional file 3.

### General organization of information used for analyses

There are 6,383 BRs represented within the 25,764 pair-wise comparisons examining binding differences across eight different individuals (NA18526, NA19099, NA12892, NA18951, NA18505, NA12878, NA12891 and NA10847) and these were previously established in the study encompassing multiple RELA-ChIP experiments [6]. Genomic information for the eight individuals (April 2009 release) was obtained from a database maintained as part of the '1000 Genomes' project [48].

### Analysis of trait/disease associated SNPs in relation to *in vitro *and *in vivo *data

The National Human Genome Research Institute has created a comprehensive database of all GWAS publications, which must assay over 100,000 SNPs in the initial stage to be included [49]. Also, only SNPs with *P *< 10e-5 are reported in the database. This database was accessed and available content downloaded on 15 November 2010. All 3407 TASs in the database were mapped to the nearest BRS and ordered according to distance of the TAS to the center of the peak summit. Figure 7 shows examination of the TAS together with the SNP(s) under the peak that leads to changes in the *in vitro *binding affinity in the EMSA-seq data. Genotypic information was obtained for these TASs for all eight individuals in the study using the hapmap database [50]; this contains all eight individuals in their genotyped cohorts.

## Abbreviations

bp: base pair; BR: binding region; BRS: binding region summit; ChIP-Seq: chromatin immunoprecipitation-sequencing; EMSA-Seq: electrophoretic mobility shirt assay-sequencing; GEO: Gene Expression Omnibus; GWAS: genome-wide association study; NF: nuclear factor; PCR: polymerase chain reaction; PWM: position weight matrix; SELEX: systematic evolution of ligands by exponential enrichment; SNP: single nucleotide polymorphism; TAS: trait/disease-associated SNP; TF: transcription factor; TFBS: transcription factor binding site.

## Competing interests

The authors declare that they have no competing interests.

## Authors' contributions

DW and AT did experimental design, performed experiments, analyzed data and wrote the manuscript. SO performed data analysis. PH did statistical analysis. INL, DA and SD did auxiliary experimental work and interpretation of data. DS supplied material. MLB and TS supplied auxiliary data and contributed to discussions. IAU conceived the study, supplied material and contributed to the writing of the manuscript. JR conceived and coordinated the study, and contributed to the writing of the manuscript. All authors have read and approved the final manuscript.

## Supplementary Material

Additional file 1 **Supplementary figures, tables (with legends) and documentation**. Click here for file

Additional file 2 **Dataset for nine NF-κB dimers (protein-binding microarrays)**. Click here for file

Additional file 3 **Information for canonical sequences of NF-κB**. Click here for file

Additional file 4 **Information from GWASs used in our analyses**. Click here for file

Additional file 5 **Sequences of primers and oligonucleotides used in this study**. Click here for file

Additional file 6 **Complete probe-specifications for protein-binding microarrays in this study**. Click here for file

Additional file 7 **MATCH scores for all 11-mer sequences derived using both reference and alternative binding models**. Click here for file
