# Supplementary material for: Extensive characterization of NF-κB binding uncovers non-canonical motifs and advances the interpretation of genetic functional traits
Source: Genome Biol. 2011 Jul 29;12(7):R70. doi: 10.1186/gb-2011-12-7-r70 (PMC3218832; doi:10.1186/gb-2011-12-7-r70)
Supplement: Additional file 1 — Supplementary figures, tables (with legends) and documentation. [file gb-2011-12-7-r70-S1.PDF]

SUPPLEMENTARY MATERIAL

Microarrays (PBMs)

Description of probe-design on the microarrays. Our microarrays are chips of 8 arrays each with over 15000 probes per array. Each array contains 577 manufacturer-probes (Agilent) and 15154 customized probes. Each probe is represented using 4 different flanks of 4-nt length: AGCT, ATGA, AGTC, AGAT and each flanked probe is replicated 4 times. Additional File 6, "probe_design_microarray.txt" shows a breakdown of the number and type of probes present on each array.

Protocol for generation and use of double-stranded protein microarrays. Single stranded probes on each array were rendered double-stranded with the following procedure. For each array on a 8x15K chip, 820 µl of "ds-mix" (NEB buffer 2, 0.1 µM dsPrimer, 2.5 X BSA, 163 µM dNTPs, 1.63 µM of Cy3-dCTP and 27.2 U of Klenow DNA polymerase I) was dispensed onto a "1x205K gasket", combined with a chip, the entire unit sealed within a hybridization chamber and incubated within a rotating-oven at 37 ºC for 90 min. The following washes were then carried out: 6 washes in 0.01 % Triton-X/PBS for 3 min each followed by a 3 min wash in PBS. Arrays were dried via centrifugation. To ascertain overall success of the procedure, arrays were scanned using the Agilent Microarray Scanner at maximum power and the image analyzed for extent of Cy3-incorporation within individual probes.

Prior to hybridisation, arrays were blocked, washed according to manufacturer's guidelines and incubated in 2 % milk/PBS for 1 h at room temperature. This was followed by 2 washes (6 min each; 0.1% Tween-20/PBS followed by 0.01% Triton X-100/PBS) and ended with a brief rinse in water before drying via centrifugation. Hybridizations were performed using a protein concentration of 0.02 µg/µl in 45 µl of protein binding reaction mix (12 mM HEPES pH 7.8, 100 mM KCl, 1 mM EDTA, 12 % glycerol, 2.8 µg poly dI-dC [Sigma P4929] and 2 % milk). Protein binding reaction mixes were dispensed into the different compartments of an 8x15K gasket slide (Agilent), combined with a chip and the entire unit sealed into a hybridization chamber. The assembled unit was rotated in the hybridization oven for 1 h at room temperature. Arrays were then subsequently washed 6 times with 1 % Tween-20/PBS for 6 min each and a further 6 washes with 0.01 % Triton X-100/PBS for 6 min each. This was followed by a brief rinse in water and drying via centrifugation. Labelling of bound protein was carried out in two stages. Firstly, arrays were incubated with 0.8 µg of primary rabbit anti-His antibody (Santa Cruz) in a 2 % milk/PBS solution for 1 h at room temperature. This was followed by 6 washes with 0.05 % Tween-20/PBS for 3 min each and other 6 washes with 0.01 % Triton X-100/PBS for 3 min. Subsequently, arrays were incubated with 6 µg of secondary Cy5-conjugated anti-rabbit IgG antibody in a 2 % milk/PBS solution for 30 min at 37 °C before being washed as per above. Before drying, arrays were first rinsed in PBS for 6 mins and then briefly again in water.

Arrays were dried via centrifugation and scanned using the Agilent Microarray Scanner at maximum power.

EMSA-Seq

Double stranding of oligos to create double stranded ligands. Single-stranded molecules were rendered into double-stranded ligands as follows: in a 20 µl reaction, 50 µM of single stranded oligonucleotide and 40 µM of Primer 1 were buffered in NEB Buffer 3 and annealed using conditions described above for the oligos TNF-promoter (biotinylated) and "TNF-promoter

complementary". 18 µl was then transferred into a 30 µl mix comprised of 10 units Large Klenow Fragment (NEB #M0210S), 167 µM dNTPs and NEB Buffer 2. Incubation was carried out at 25°C for 30 mins followed by 37°C for 30 mins. DNA was purified using phenol-chloroform separation and concentrated via ethanol precipitation into ultrapure water.

Deep-sequencing library preparation. In this work, standard "Paired-End" adaptor-molecules as described in Illumina's ChIP-protocol and the NEBNext DNA Sample Prep kit (NEB #E6000S) were used to create libraries for deep sequencing in the Genome Analyzer (GA). 10 ng of DNA was used for each library-preparation.

EMSA, DNase I and UV laser footprinting

Preparation of labelled, double stranded probes for use during footprinting. Typically, 20 pmol of either strand from a complementary pair of oligos (MWG) were 5' end-labelled using T4 polynucleotide kinase with [γ-^{32}P]ATP. The labelled strand was annealing with two-fold excess of its complementary strand and the DNA was treated by Fpg DNA N-glycosylase to remove pre-existing oxidative guanine lesions (Angelov *et al.*, Biophys J 88, 2766-78 (2005); a kind gift from Serge Boiteux, Commissariat à l'Energie Atomique-Fontenay aux Roses, France). Labelled DNA probes were purified via denaturing gel electrophoresis and re-annealed to form double stranded molecules. Under the conditions used, 100 % of labelled probes were rendered double-stranded.

Statistical analyses

Derivation of enriched k-mer sequences for datasets generated by EMSA-Seq. Raw data in the form of reads (FASTQ format) obtained after deep sequencing has been subsequently processed using both UNIX and perl scripts. Briefly, only sequenced reads that contained matches to at least 4 bp-sequences flanking the degenerate region were considered acceptable for further processing. In addition, for reads to be acceptable the three regions on the DNA ligand (two flanks and degenerate) must not contain bases annotated as "N" and furthermore, bases must have a Phred quality score of at least 20. For ligands with 10-mer degnerate regions, a 10-mer sequence was extracted. For ligands with 20-mer degenerate region, 20-mer sequences corresponding to the degenerate segment of DNA ligands were then extracted and "lower" order 11-mer sequences derived from these using a publicly available resource, Meryl the k-mer counter. Forward and reverse-complementary orientations of all 11-mers have been condensed by Meryl into a single sequence known as a "Meryl 11-mer" with its final form of representation dependant on an ascending alphabetical basis. This "Meryl-representation" of a 11-mer is used throughout.

Analyses of enriched 11-mers

MATCH-scoring of sequences. MATCH-scores for 11-mer sequences can be found in Additional File 7, "11mer_MATCH_comparisons.txt": column1 is the 11-mer sequence, column2 is the MATCH-score based from alternative binding-model whilst column3 is the MATCH-score based on V$NFKB_Q6_01.

Binding models generated using the top affinity binders from microarrays

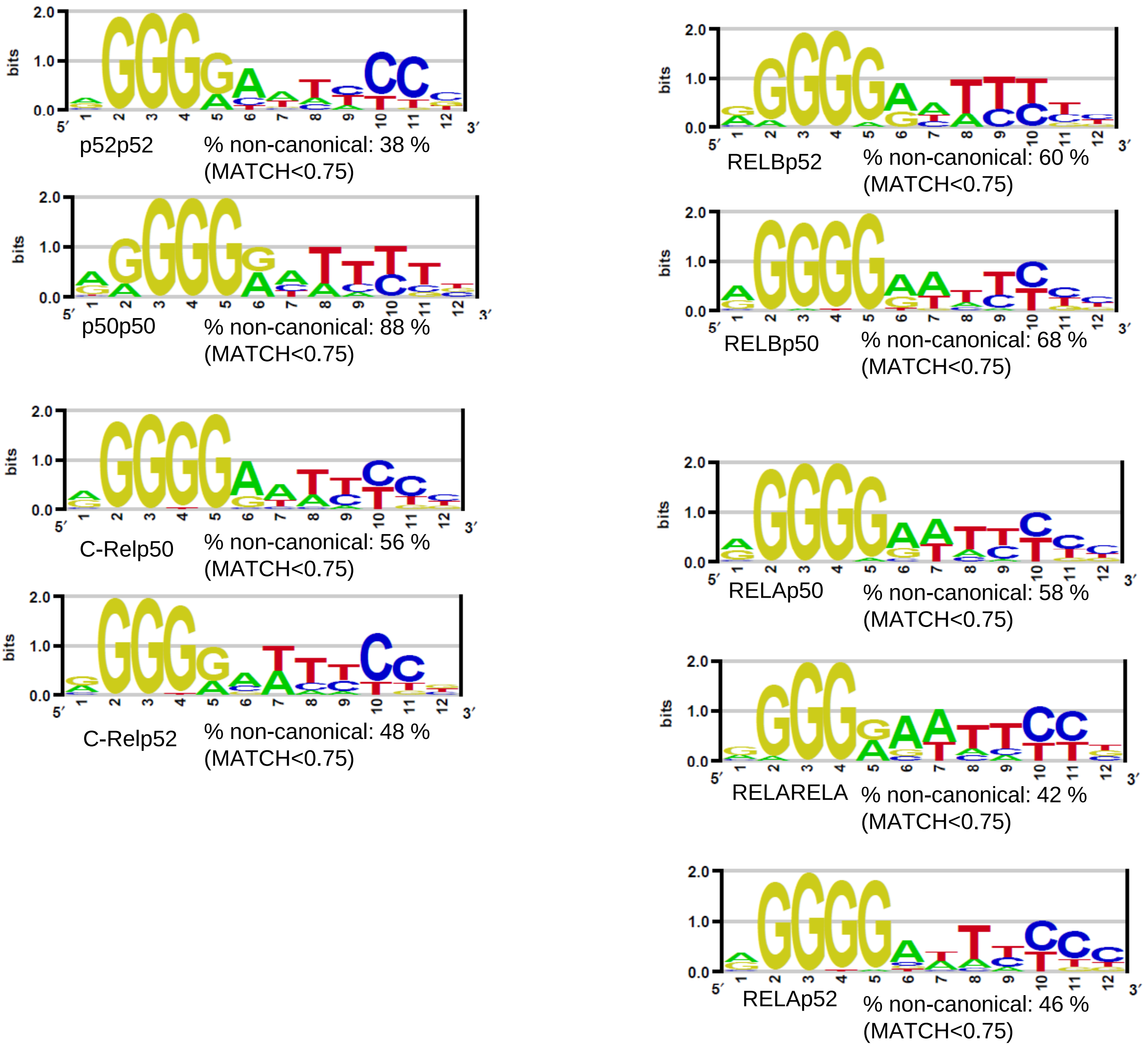

Figure 1. *De novo* motif identification was performed on the 50 top-scoring 11-mer sequences from each experiment using the Priority algorithm. No priors were used for motif identification. Logos were generated using the enoLOGOS web tool. For every dimer, the % proportion of sequences that are non-canonical (MATCH < 0.75) and that have contributed towards construction of the motif has been indicated.

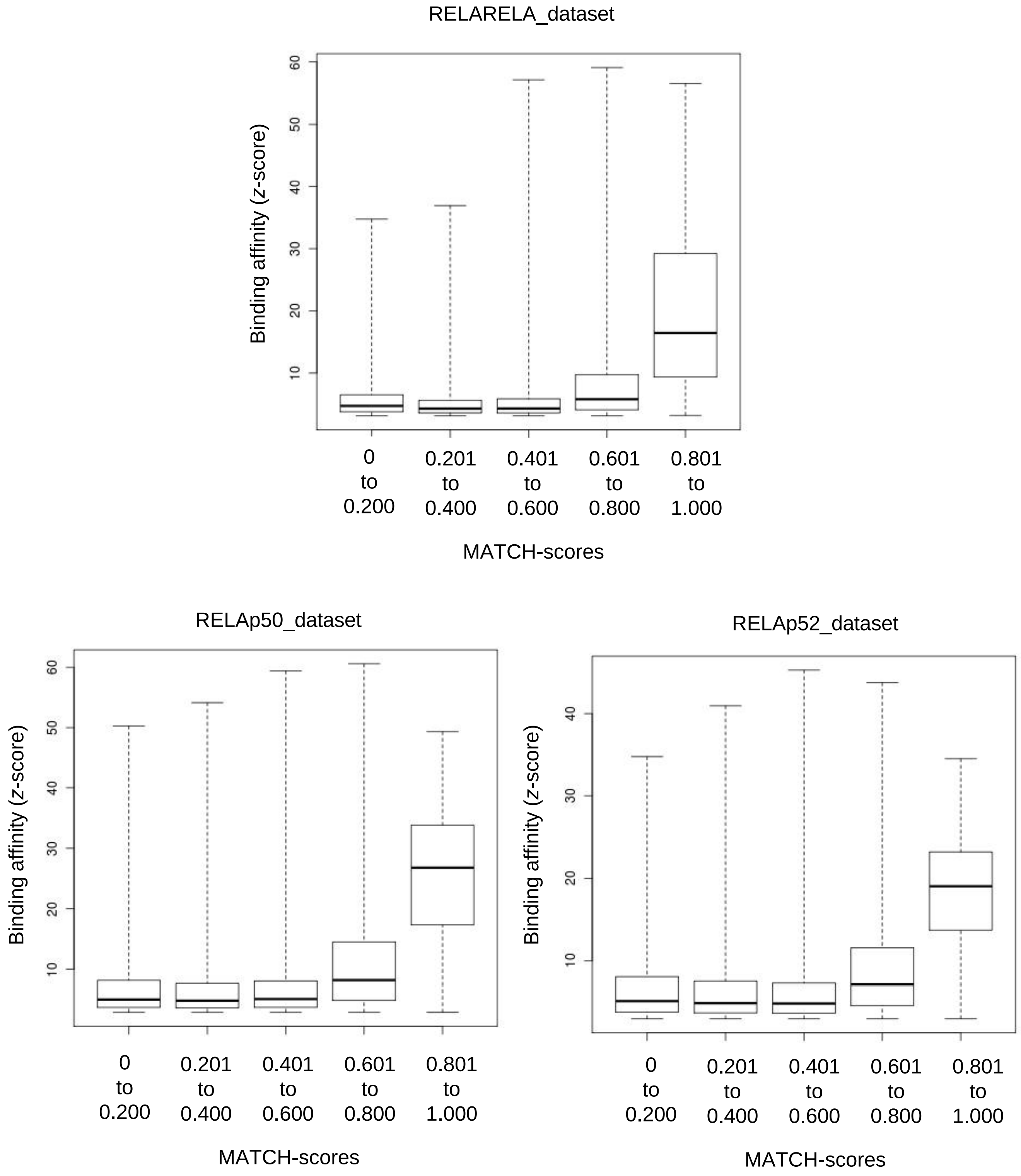

Figure 2. Groupings of 11-mers from each of the RELA-dimer enriched datasets (EMSA-Seq) formed on the basis of MATCH-score similarity to the reference binding model.

Whilst the median z-score increases with MATCH-score, there exists variation amongst 11-mers that have comparable MATCH-scores.

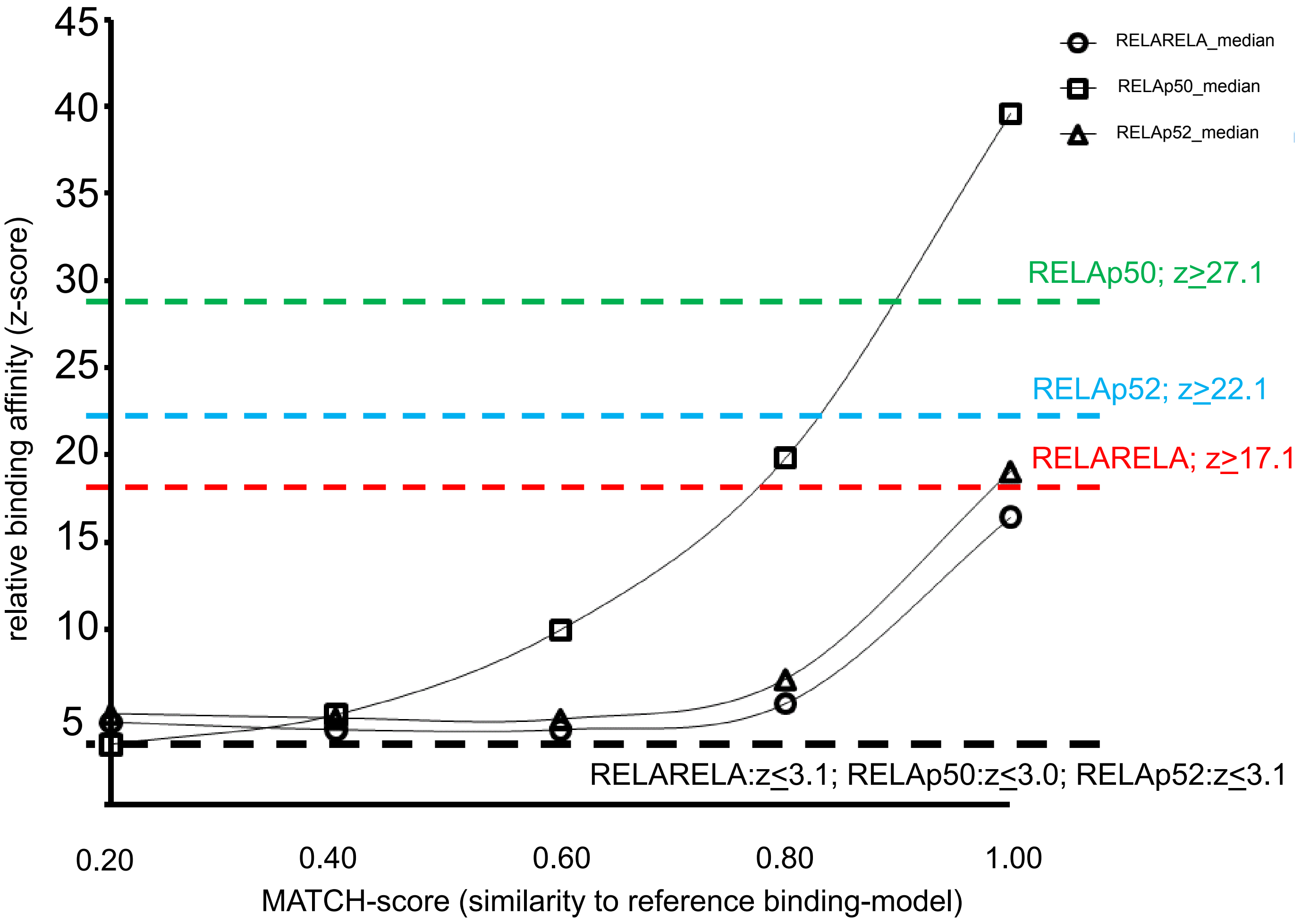

Top5000 11-mers

Total pairs/BRs	=1422 (464 BRs)	
Direct +ve binding NF-κB	=1008 (345 BRs)	70.9 % success
Other factors	=414 (172 BRs)	

Bottom5000 11-mers

Total pairs/BRs	=1442 (532 BRs)	
Direct +ve binding NF-κB	=734 (311 BRs)	50.9 % success
Other factors	=708 (307 BRs)	

Rationalizing *in vitro* binding potential from the top and bottom 5000 RELA-dimer enriched 11-mers and *in vivo* TF-binding

(a)

Figure 3a

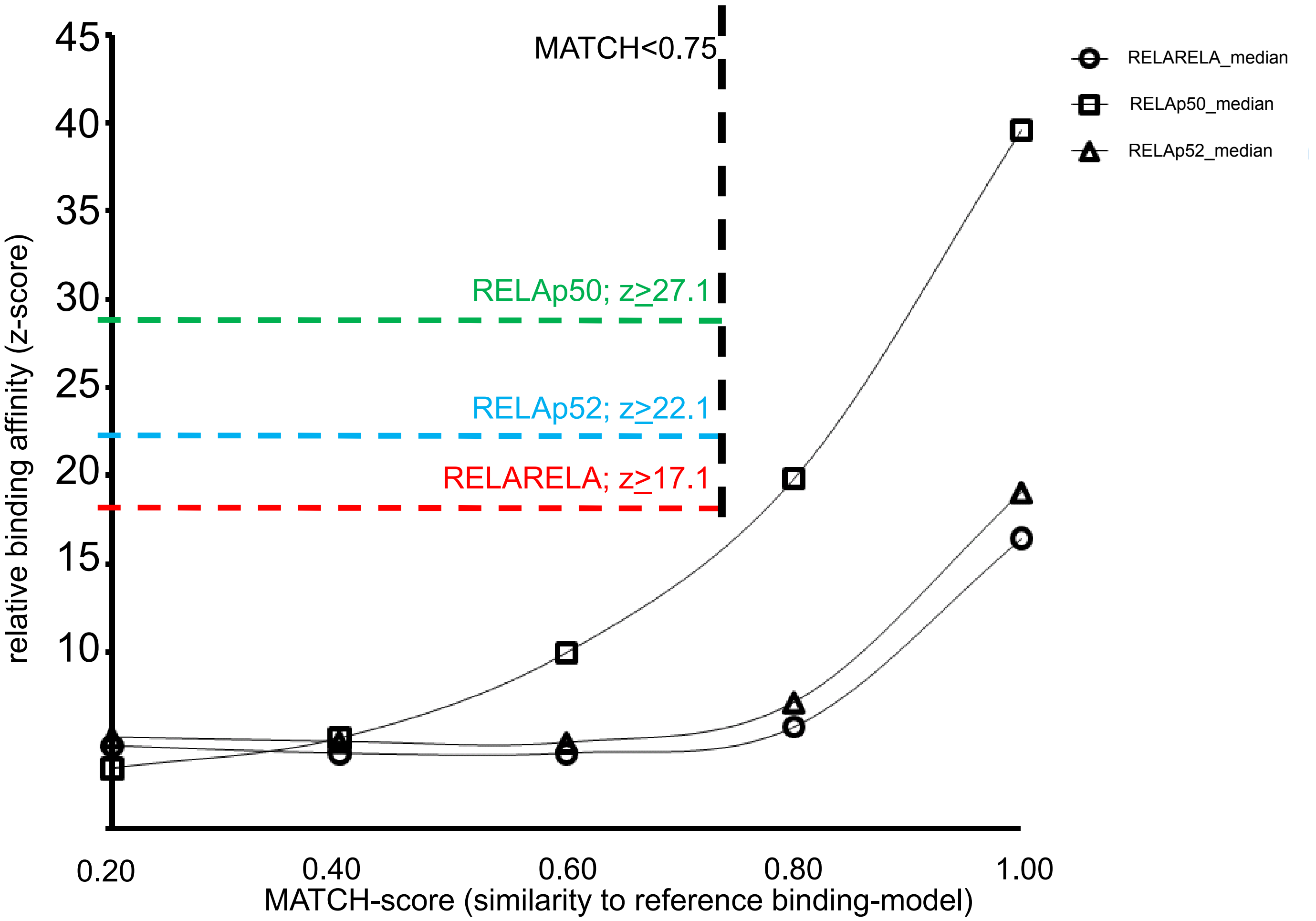

Top5000 11-mers with MATCH –scores <750

Total pairs/BRs	=1384	
Direct +ve binding NF-κB	=975	70.5 % success
Other factors	=409	

Rationalizing *in vitro* binding potential from the top 5000 RELA-dimer enriched 11-mers with MATCH-scores less than 0.75 and *in vivo* TF-binding

(b)

Figure 3b

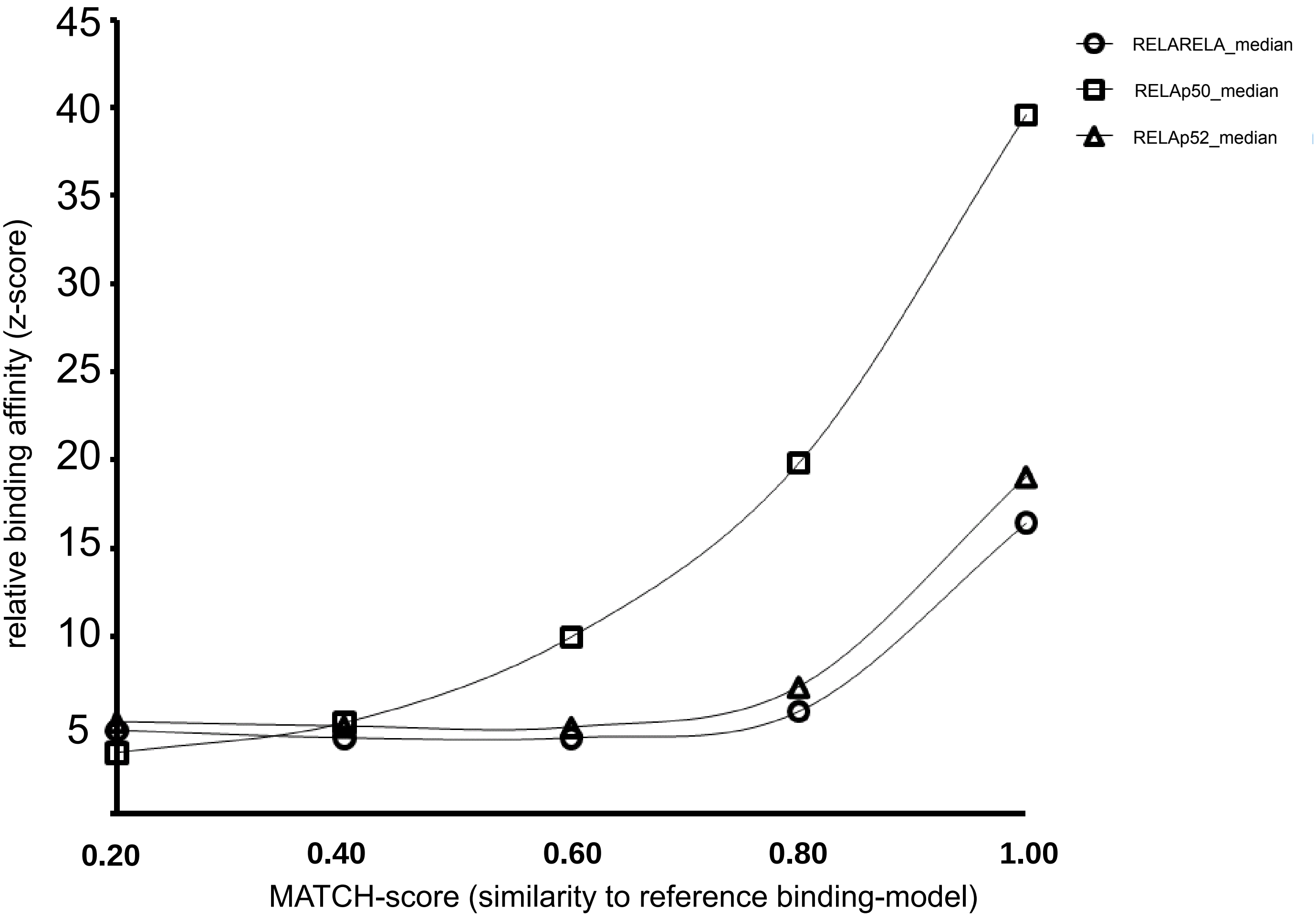

"extended NF-κB Binders" set of 11-mers

Within the five MATCH-score bins :0-0.20, 0.201-0.40, 0.401-0.60, 0.601-0.8 0 and 0.801-1.0 only 11-mers with a z-score no less than 10% below that of the median z-score for that bin were used

Total pairs/BRs	=5452 (1959 BRs)	
Direct +ve binding NF-κB	=3559 (1405 BRs)	65.3 % success
Other factors	=1893 (883 BRs)	

Rationalizing *in vitro* binding potential from selected sets of RELA-dimer enriched 11-mers

(c)

Figure 3c

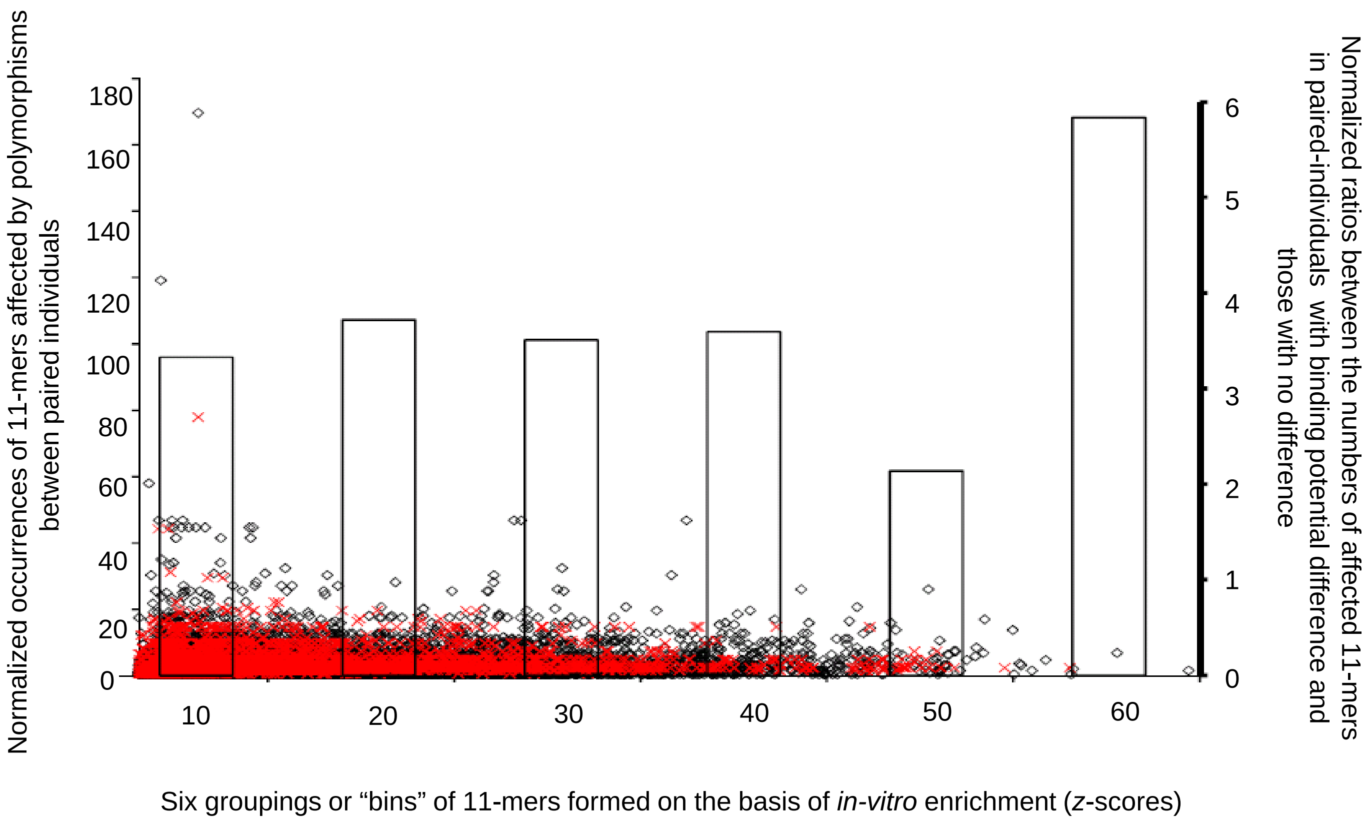

Figure 4. Comparison of enriched 11-mers in paired-individuals with polymorphisms in the BRS (pairs with difference in NF-kB binding potential versus those with no apparent difference).

Normalized number of occurrences for an affected 11-mer between paired-individuals (left-side vertical axis) is shown for pairs with a binding potential difference (open, **black** circles) versus those without (**red** crosses).

The numbers of affected 11-mers between paired-individuals with a binding potential difference versus those without a difference were expressed as normalized ratios within 6 "bins" based on z-scores: 0-10, 10-20, 20-30, 30-40, 40-50 and 50-60 (hollow bars; right-side vertical axis).

Datasets	RELARELA	RELAp50	RELAp52
Number of 11-mer sequences in common between microarrays and EMSA-Seq	240	446	475
Pearson-test of ranked affinities (correlation)	0.77	0.77	0.84

Figure 5. Ranked affinities were determined using the individual binding affinities (*z*-scores) for 11-mer sequences within both the microarray and EMSA-Seq datasets.

These ranked affinities were then compared to ascertain overall similarity for sequences tested on both platforms.

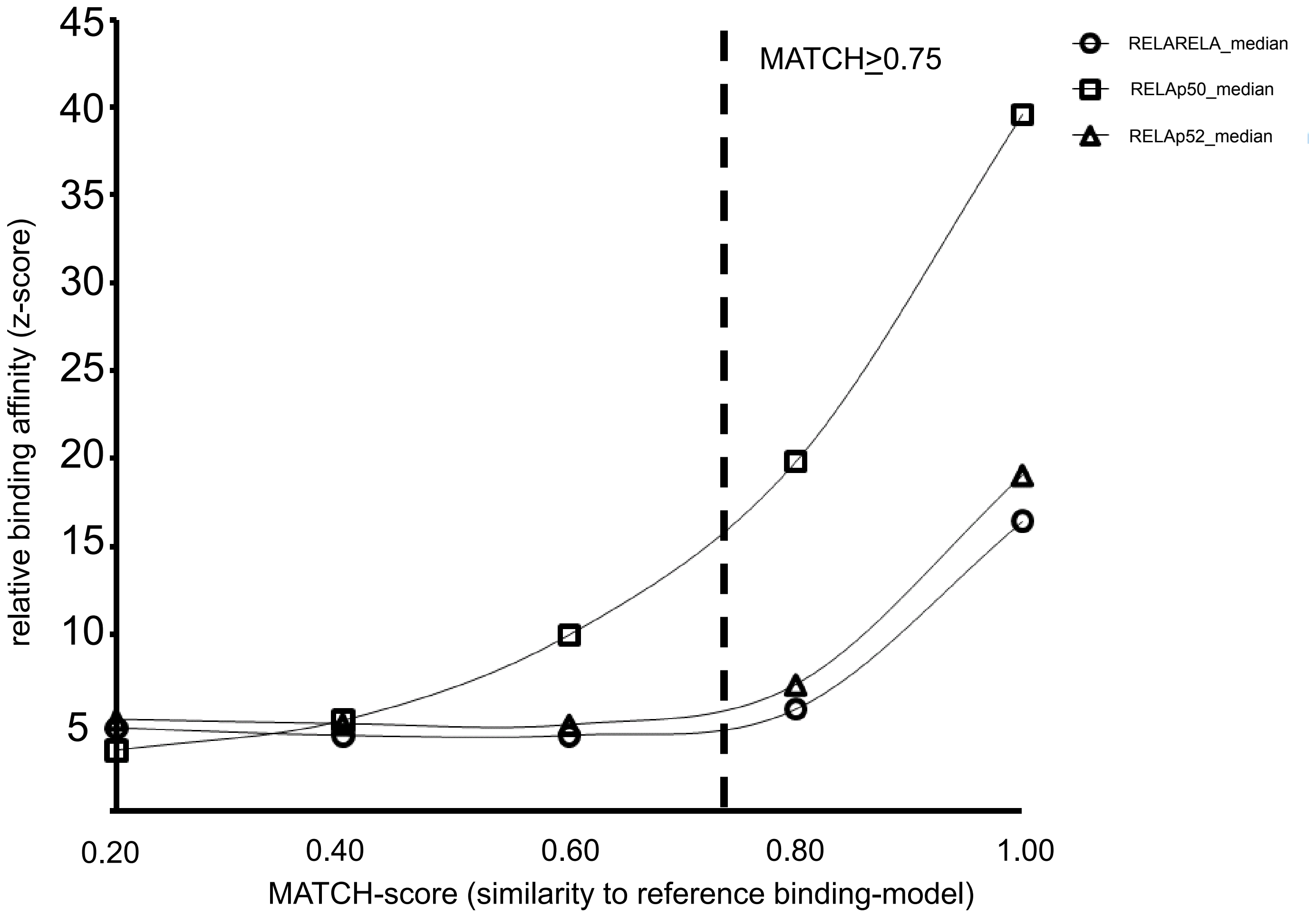

"canonical NF-κB Binders" set of 11-mers with MATCH-scores ≥0.75

Total pairs/BRs	=892 (276 BRs)	
Direct +ve binding NF-κB	=730 (222 BRs)	81.8% success
Other factors	=162 (80 BRs)	

Rationalizing *in vitro* binding potential from RELA-dimer enriched 11-mers having MATCH-scores ≥0.75 with *in vivo* TF-binding

Figure 6

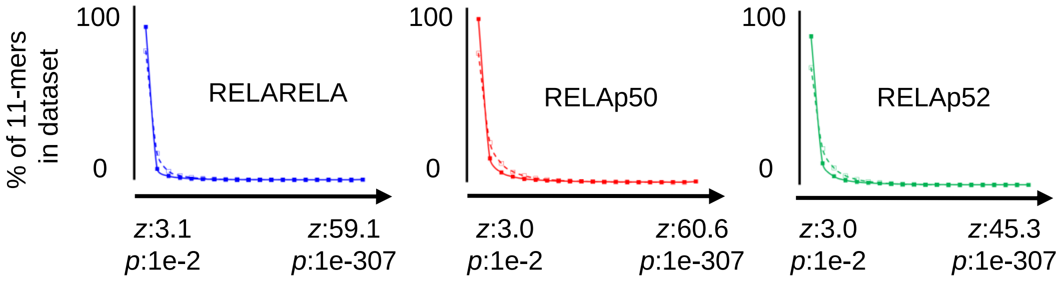

Figure 7. Distribution of p-values (solid lines) and z-scores (broken lines) for sequences enriched during EMSA-Seq.

From left to right on the horizontal axes, these correspond to increasing binding affinities of the RELA-containing dimers for 11-mers. If an 11-mer was highly enriched after selection (z-score) with a corrected p-value after multiple testing of less than 0.01, it was considered to be a binder. Z-scores were used as a proxy for relative affinities, and for each individual dimer ranked affinities derived using z-scores were in full agreement with the ranked affinities from p-values (correlation coefficient 1; Pearson-correlation test).

This is a robust indicator that our statistical model has correctly identified the highly enriched sequences to also be high confidence-candidates (datasets uploaded into the GEO database with acc# GSE29460).

RELBp50				C-Relp52				RELBp52			
AGCT	0.986			AGCT	0.967			AGCT	0.978		
AGTC	0.973	0.976		AGTC	0.963	0.965		AGTC	0.967	0.970	
ATGA	0.985	0.984	0.977	ATGA	0.968	0.969	0.966	ATGA	0.975	0.975	0.965
	AGAT	AGCT	AGTC		AGAT	AGCT	AGTC		AGAT	AGCT	AGTC
p50p50				C-Relp50				p52p52			
AGCT	0.972			AGCT	0.983			AGCT	0.984		
AGTC	0.963	0.970		AGTC	0.975	0.974		AGTC	0.979	0.982	
ATGA	0.955	0.958	0.947	ATGA	0.977	0.977	0.970	ATGA	0.986	0.981	0.979
	AGAT	AGCT	AGTC		AGAT	AGCT	AGTC		AGAT	AGCT	AGTC
RELARELA				RELAp52				RELAp50			
AGCT	0.936			AGCT	0.981			AGCT	0.979		
AGTC	0.900	0.885		AGTC	0.973	0.979		AGTC	0.745	0.735	
ATGA	0.922	0.917	0.888	ATGA	0.979	0.977	0.974	ATGA	0.743	0.731	0.969
	AGAT	AGCT	AGTC		AGAT	AGCT	AGTC		AGAT	AGCT	AGTC

Table 1. Pair-wise comparisons of z-scores for the 803 microarray-probes done between flank-specific datasets within dimers. Numbers shown are similarity coefficients from Pearson-correlation tests.

p5 0	0.90							
RELA	0.76	0.57						
RELBp52	0.95	0.90	0.66					
RELBp50	0.95	0.91	0.64	0.97				
p5 2	0.92	0.82	0.73	0.94	0.92			
RELAp52	0.97	0.88	0.74	0.97	0.95	0.97		
C-Relp50	0.96	0.91	0.76	0.93	0.93	0.88	0.94	
C-Relp52	0.96	0.84	0.79	0.95	0.92	0.96	0.98	0.94
	RELAp50	p5 0	RELA	RELBp52	RELBp50	p5 2	RELAp52	C-Relp50

Table 2. Pair-wise comparisons of microarray-derived binding affinities (z-scores) across NF-κB dimers (similarity coefficient from Pearson correlation test).

For example, the dimer RelARelA has a binding profile that is distinctively different from those of the other eight dimers (average coefficient of 0.71, with highest being 0.79 and lowest at 0.57; Pearson-correlation tests).

reference binding-model

nucleotide_position	A	C	G	T
1	0.107	0.120	0.467	0.307
2	0.040	0.027	0.907	0.027
3	0.053	0.013	0.893	0.040
4	0.613	0.001	0.360	0.027
5	0.827	0.040	0.120	0.013
6	0.947	0.027	0.027	0.001
7	0.213	0.147	0.293	0.347
8	0.053	0.200	0.027	0.720
9	0.040	0.905	0.001	0.053
10	0.013	0.959	0.001	0.027
11	0.133	0.773	0.013	0.080
V$NFKB_Q6_01 (TRANSFAC)				

alternative

nucleotide_position	A	C	G	T
1	0.108	0.245	0.446	0.202
2	0.005	0.005	0.985	0.005
3	0.000	0.000	1.000	0.000
4	0.893	0.000	0.107	0.000
5	0.993	0.000	0.007	0.000
6	0.823	0.000	0.030	0.147
7	0.097	0.278	0.294	0.331
8	0.010	0.318	0.050	0.622
9	0.164	0.836	0.000	0.000
10	0.005	0.983	0.006	0.005
11	0.199	0.435	0.258	0.108
RelA_dimers EMSA_Seq				

Table 3
